# Angelica Stem: A Potential Low-Cost Source of Bioactive Phthalides and Phytosterols

**DOI:** 10.3390/molecules23123065

**Published:** 2018-11-23

**Authors:** Chengke Zhao, Yuan Jia, Fachuang Lu

**Affiliations:** 1 State Key Laboratory of Pulp and Paper Engineering, South China University of Technology, Guangzhou 510640, China; mszck@foxmail.com (C.Z.); 201620121278@mail.scut.edu.cn (Y.J.); 2 Guangdong Engineering Research Center for Green Fine Chemicals, Guangzhou 510640, China

**Keywords:** *Angelica sinensis*, bioactive compounds, GC–MS, phthalides, coniferyl ferulate, phytosterols

## Abstract

Chinese Angelica is a significant medical plant due to the various therapeutic constituents in its root; whereas the aerial part is considered worthless and often discarded as agricultural waste. In this work, phytochemicals from the stem were first systematically analyzed by means of GC–MS after derivatization and HPLC–MS/MS in multiple reaction monitoring (MRM) mode. Phthalides, ferulic acid, and coniferyl ferulate were detected in the stem; although their content is relatively low in comparison with the root. Some specific compounds, such as p-hydroxybenzoic acid, vanillic acid, protocatechuic acid, caffeic acid, 4-hydroxyphenyl-1, 2-ethanediol, thymol-*β*-d-glucopyranoside, etc. and a significant amount of phytosterols (1.36 mg/g stem, mainly β-sitosterol) were detected in the stem. The extracted oil from the stem contained a considerable amount of phthalides (48.5 mg/g), *β*-sitosterol (56.21 mg/g), and stigmasterol (14.03 mg/g); no other bioactive compounds were found that could be potentially used as pharmaceuticals or additives to healthcare food.

## 1. Introduction

Medicinal plants are valuable and sustainable sources of therapeutic agents and health-care foods. *Angelica sinensis* (AS) is a perennial Apiaceae plant [1,2]. Its root (also named *Radix Angelica sinensis*) is a traditional and ethnologic medicine in China, and has long been employed for tonifying and invigorating blood, lubricating the intestines, relieving pain, as well as treating female irregular menstruation and amenorrhea [3,4]. Also, AS root is used as a dietary supplement for women’s care in Asia, Europe, and America. Great efforts have been directed towards the phytochemistry of the AS root. The main bioactive constituents include phthalides, ferulic acid, and coniferyl ferulate [3,4]. *Z*/*E*-ligustilides and n-butylidenephthalides in apiaceae are active ingredients that can reduce platelet aggregation, enhance microcirculation, inhibit uterine contractions, and also exert analgesic, anti-inflammatory, antiproliferative, and antifungal effects [5,6,7,8]. Ferulic acid has antioxidant, anti-inflammatory, and anti-Alzheimer’s functionalities [9,10,11,12]. Coniferyl ferulate exhibits bioactivities, such as antibacterial, antioxidant, anticancer, and an inhibitory effect towards the progression of tetrachloromethane (CCl_4_)-induced liver fibrosis [13,14,15,16]. The concentrations of these compounds in Angelica vary with its species, geographic sources, harvesting time, and processing methods, which all influence its therapeutic activities [3,4].

Despite the multiple medical functions of the root, a long-time interval (normally three years) is needed for its growth in order for it to be used as a high-quality medical material. The commercial AS root is expensive, and usually supplied in the form of slices, powders, or dispensing granules as a health food or therapeutic medicine [17]. It is impractical to use the root as a feedstock to extract bioactive compounds for pharmacological applications or to further purify it for medicinal synthesis. However, the aerial part, mainly the stem, is abundant, and potentially can be used as a low-cost feedstock to isolate bioactive ingredients for medical applications or health-care products. Actually, it is usually regarded as agricultural waste to burn it or allow it to decompose on the ground. Although Zhou and coworkers have isolated some bioactive compounds (including 24, 24-dimethyl-9,19-cyclolanostan-3*β*-ol, uracil, hyperoside, allantoin, 1-(4-hydroxyphenyl)-1, 2-ethanediol, etc.) from the aerial part [18], there is a lack of effort focusing on the comprehensive identification of the constituents and quantitative analysis of bioactive compounds in the AS stem. Moreover, the differences of the bioactive constituents in the stem and root lack understanding.

**Figure 1:**
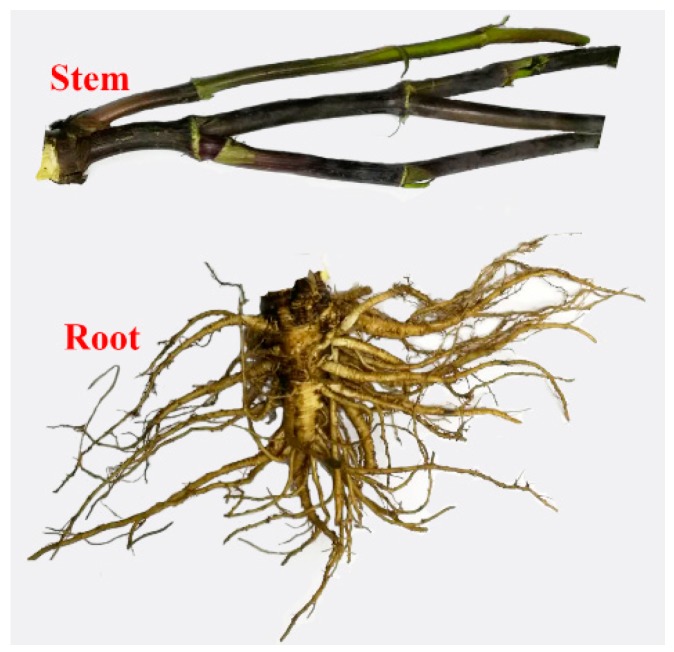
Photographs of *Angelica sinensis* stem and root.

In the work, the fresh Angelica plant was divided into two parts (root and stem, Figure 1), the constituents in the oily extract of the AS stem were comprehensively analyzed by gas chromatography–mass spectrometry (GC–MS) after trimethylsilyl derivatization. Ferulic acid and chemically unstable coniferyl ferulate were concurrently monitored by a triple-quadrupole high-performance liquid chromatography–mass spectrometer (HPLC–MS/MS) on multiple reaction monitoring (MRM) mode. The significant 16 bioactive compounds in the stem, mainly including phthalides, some organic acids, phytosterols, and coniferyl ferulate, were quantified with the aid of standard compounds, and compared with the compounds from the root. The objectives of our work are (1) to provide some phytochemical information about the medical AS plant and (2) to explore an abundant and low-cost source of some bioactive compounds for pharmacy or healthcare food applications.

## 2. Results and Discussion

### 2.1. Extraction and Purification of Extracts

In the work, optimized ultrasonic extraction using methanol-formic acid (95:5, *v*/*v*) as a solvent at a temperature 30 °C was applied for the extraction of organic compounds from AS stem and root samples [19]. Introduction of formic acid in methanol can facilitate the extraction efficiency of coniferyl ferulate, ferulic acid, ligustilide, and butylidenephthalide. Mild temperature extraction can avoid the degradation and conversion reactions of unstable coniferyl ferulate and *Z*-ligustilide [19,20]. Ultrasonic extraction can result in dissolution of some carbohydrates in the solvent. The carbohydrates were removed by (1) one-step precipitation with absolute methanol and then (2) aqueous extraction in ethyl acetate. The organic phase was dried and the purified oily extract was obtained. The yields of oily extracts for the AS stem and root were 1.8% and 3.3%, respectively.

### 2.2. GC/MS Analysis

#### 2.2.1. Identification of Phytochemicals

Since many compounds with hydroxyl and carboxyl groups in AS extracts are difficult to detect using GC–MS, trimethylsilyl derivatization can decrease the polarities of compounds making them easier to detect. The chemical structures of constituents were identified based on the Shimadzu Mass Spectra Library (similarity ≥88%) or with the aid of standard compounds.

**Figure 2:**
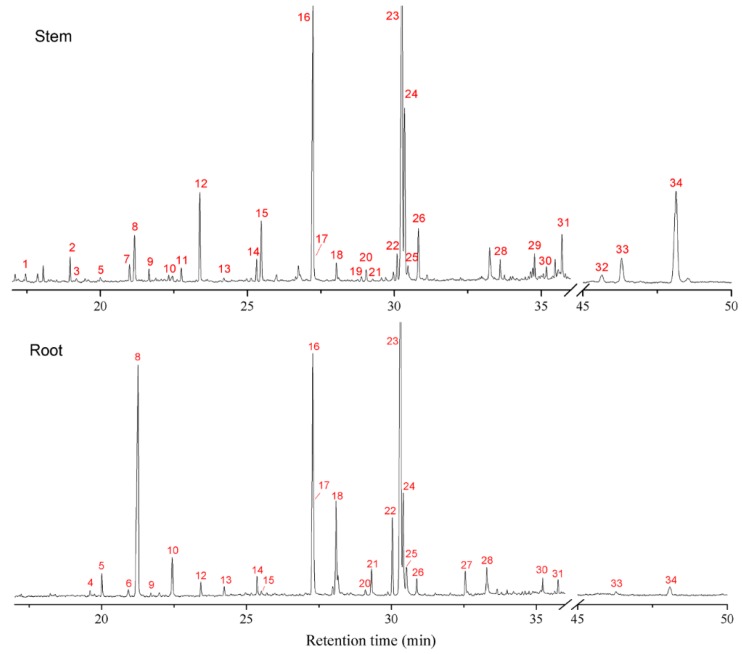
Typical GC chromatograms of TMS-derivatized oily extracts from AS stem and root. The identified compounds were numbered according to retention time. Peak labels are the same as the numbers in Figure 3 and Table 1.

**Figure 3:**
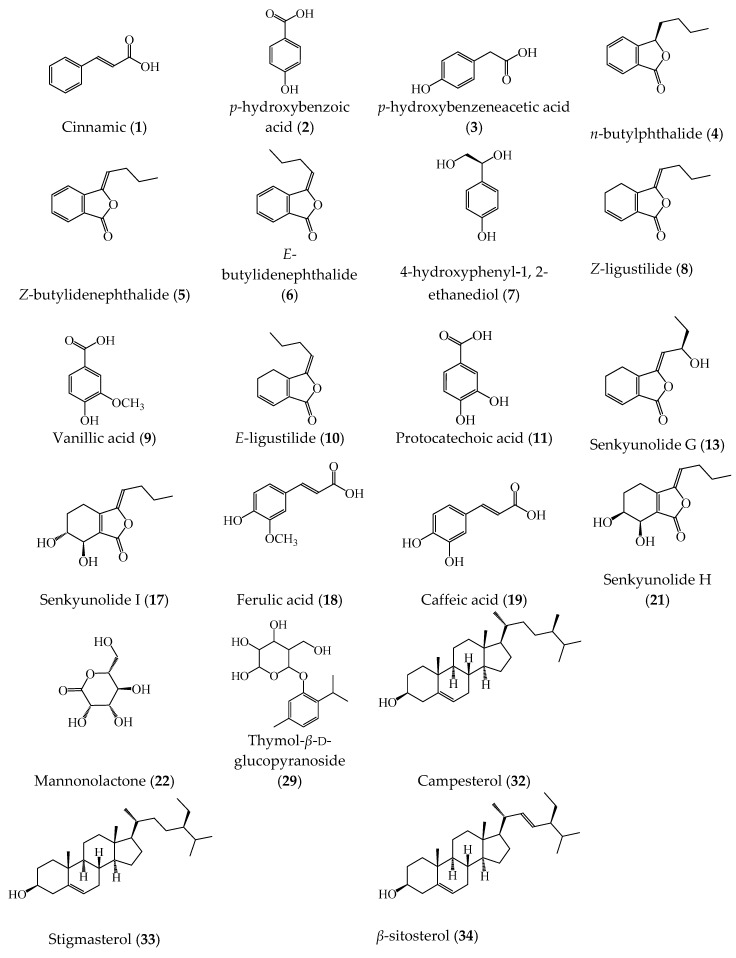
Chemical structures of some compounds identified in extracts of *Angelica sinensis* stem and root by GC–MS. The numbers in brackets correspond to the peak numbers of GC chromatograms in Figure 2.

The typical GC spectra of AS stem and root extracts after trimethylsilyl derivatization are shown in Figure 2. The identified compounds from the spectra are listed in Table 1, and the structures of some important bioactive compounds are shown in Figure 3. From the GC spectra, 34 compounds were tentatively identified from the extracts, mainly including phthalides, organic acids, and phytosterols. Phthalides are a kind of significant compounds in the AS root, and are responsible for many bioactivities [4]. Butylphthalide (4), *Z*/*E*-butylidenephthalide (5/6), *Z*/*E*-ligustilide (8/10), and Senkyunolide G, I, H (13, 17, 21) are the main phthalides that can be identified in the chromatogram of AS root extract. Whereas, only *Z*/*E*-ligustilide, Senkyunolide I, and *Z*-butylidenephthalide can be detected in the stem extract. Senkyunolide I (17) was identified by comparing the retention time and mass information with a standard compound; though its peak partially overlaps on the peak of palmitic acid. Recently, ligustilide dimers (riligustilide and levistolide A) and trimers (triligustilide A and B) from AS root were isolated and recognized [21,22]. These compounds can also inhibit platelet aggregation, although polymerization can weaken the activity [22]. These compounds were not identified from the GC–MS analysis, presumably due to their very low content [21].

Long chain organic acids, including palmitic acid (16), linoelaidic acid (23), and *Z*-oleic acid (24) are the main constituents in the extracts of both stem and root. Ferulic acid (18) is abundant in the AS root extract and also can be identified in the stem extract. Other organic acids with a benzene ring, such as cinnamic acid (1), *p*-hydroxybenzoic acid (2), *p*-hydroxybenzeneacetic acid (3), vanillic acid (9), protocatechuic acid (11), and caffeic acid (19) were detectable in the stem extract, whereas they were not found in the root extract. In phytochemistry, cinnamic and caffeic acids are significant intermediates for the biosynthesis of lignin monomers, which are related to the high level of lignification in the plant stem [23]. Vanillic acid has a hepatoprotective effect and can suppress hepatic fibrosis in chronic injury [24,25]. Protocatechuic and caffeic acids have antioxidant, anti-inflammatory, anti-glycation, and chemopreventive effects [26,27]. In addition, 4-hydroxyphenyl-1, 2-ethanediol (7), and thymol-*β*-d-glucopyranoside (29) were identified from the stem extract, whereas they were not found in the root extract. 4-hydroxyphenyl-1,2-ethanediol was also isolated from the aerial parts of AS previously [18]. Thymol-*β*-d-glucopyranoside is firstly identified in the Angelica plant; both were reported to exhibit antibacterial activity [18,28].

Phytosterols, such as campesterol, stigmasterol, and *β*-Sitosterol predominate in higher plants and some typical diets (nuts, seeds, etc.) [29]. They have been shown to have many functions, such as reducing blood cholesterol, inhibiting growth of cancer cells, enhancing immune function, and anti-osteoarthritic effects [30,31,32,33]. Phytosterols are rarely reported in the AS root, while *β*-sitosterol was previously isolated from the mixture of AS stem and leaf [18]. In the chromatogram, three phytosterols—campesterol, stigmasterol, and *β*-sitosterol—were obviously identified as the peak No. 32, 33, and 34, respectively, of the stem extract, while only stigmasterol and *β*-sitosterol were identified from the root extract with much low concentration (campesterol was not identified, probably due to the very low concentration).

Some antibacterial compounds, such as 24, 24-dimethyl-9,19-cyclolanostan-3 *β*-ol, daucosterol, allantoin, and D-mannitol were reported to exist in the aerial part [18]; whereas, they were not identified in the extract of stem. Potentially, these compounds mainly are distributed in the leaves.

#### 2.2.2. Quantitative Analysis

The contents of 15 bioactive compounds in the extracts and dry materials were quantified with aids of standard compounds. The calibration curves were obtained by plotting peak areas versus corresponding concentration of compounds (Figure S2, in supplementary materials). The results are shown in Table 2.

The content of total detectable phthalides in the root was 4.57 mg/g, including *Z*-ligustilide 2.08 mg/g, senkyunolide I 1.13 mg/g, senkyunolide H 0.58 mg/g, *E*-ligustilide 0.36 mg/g, *Z*-butylidenephthalide 0.26 mg/g, and *E*-butylidenephthalide 0.16 mg/g. The content of phthalides in the stem was 0.87 mg/g, lower than that the root. In the stem, the phthalides mainly include *Z*-ligustilide (0.51 mg/g) and senkyunolide I (0.21 mg/g). The concentration of ferulic acid in AS root was reported to be in the range of 0.211 to 1.75 mg/g as determined by the liquid chromatogram method [4]. In this work, the content of ferulic acid in root determined by GC–MS after derivatization was also in this range (0.60 mg/g); whereas its content in the stem was much lower (0.13 mg/g). The specific bioactive components in the stem included cinnamic, *p*-hydroxybenzoic, vanillic, protocatechuic, and caffeic acids. The contents of these compounds are relatively low, in range of 0.018 to 0.054 mg/g. Noticeably, AS stem contains a significant amount of phytosterols (including 1.01 mg/g *β*-sitosterol, 0.26 mg/g stigmasterol, and 0.088 mg/g campesterol). However, these compounds are very few in the root part.

After removal of organic solvent, the concentrations of bioactive compounds in the concentrated oily extract of stem were quantified (Table 2): *Z*/*E*-ligustilide (32.76 mg/g), senkyunolide I (11.5 mg/g), *Z*-butylidenephthalide (4.2 mg/g), *p*-hydroxybenzoic acid (3.48 mg/g), protocatechuic acid (3 mg/g), caffeic acid (2 mg/g), ferulic acid (7.1 mg/g), campesterol (4.93 mg/g), stigmasterol (14.03 mg/g), and *β*-sitosterol (56.21 mg/g). Other components identified from the stem extract are main long chain organic acids that have no biological toxicities. In consideration of various bioactive and pharmacological functions as well as the collaborative effects of these compounds, the extracts can be directly used for pharmaceuticals or as a health-care additive in food. In addition, due to the abundance of ligustilides and *β*-sitosterol, the oily extract from AS stem can be further used for isolation monomeric phthalides and phytosterols for drug exploitation.

### 2.3. Coniferyl Ferulate Analysis by HPLC–MS/MS

Coniferyl ferulate cannot be detected by GC–MS as it is quite unstable and tends to decompose under alkaline or heated conditions. Normally, it is analyzed and quantified by liquid chromatography [34,35]. Liquid chromatography with tandem mass spectrometry can provide high mass resolution and abundant fragment ion information of organic compounds, and is therefore adept at qualitative elucidation. The multiple reaction monitoring (MRM) mode was adopted by selectively screening insignificant ions and permitting the access of target ions for improving quantitative performance. The parameters of MRM mode were optimized prior to coniferyl ferulate determination. The mass information of coniferyl ferulate (CA-FA) is shown in Figure S3a. The molecular ions of [M + H]^+^ and [M − H]^−^ and some adduct ions are very rare in the MS spectrum, while the main daughter ions [FA − H]^−^ (*m*/*z* 193) and [CA − OH]^+^ (*m*/*z* 163) are abundant in MS spectrum. The most abundant characteristic daughter ion [FA − H]^−^ was selected for fragmentation to obtain the fragment information and corresponding optimal collision energy (CE). The optimized fragment ions and CE are shown in Figure S3b. The [FA − H]^−^ ion can produce three negative fragment ions with *m*/*z* of 178.2, 134.1, and 133.1, corresponding to the CE value of 14 v, 14 v, and 25 v.

**Figure 4:**
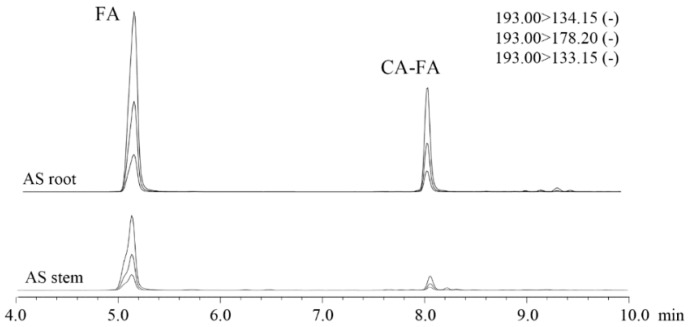
Mass Multiple Reaction Monitoring (MS–MRM) analysis of coniferyl ferulate (CA-FA) and ferulic acid (FA) in extracts of AS stem and root.

The extracts were dissolved in methanol solution with concentration of 100 ppm, and then filtrated with a 0.22-μm membrane. The solution was immediately subjected to HPLC–MRM/MS analysis by adopting optimized parameters. As the [FA − H]^−^ (*m*/*z* 193) ion can be commonly produced from ferulic acid and coniferyl ferulate, both compounds can be observed from the chromatograms (Figure 4). The contents of coniferyl ferulate and ferulic acid in the stem are 0.021 mg/g and 0.098 mg/g, which are much lower than the corresponding values in the root (0.52 mg/g and 0.58 mg/g, respectively, Table 3). The content of ferulic acid determined by GC–MS are slightly higher than that determined by LC–MS/MS, which is potentially due to the degradation of coniferyl ferulate to generate some ferulic acid during derivatization treatment before GC–MS analysis.

Still, coniferyl ferulate tends to decompose at room temperature. Figure S5 shows the degradation of coniferyl ferulate in the methanol solution with the time (concentration 1 ppm, temperature 25 °C). The concentration of coniferyl ferulate decreased to 0.48 ppm at 9 h, while it was less than 0.01 ppm at 48 h. In consideration of sample drying, and time consumption on extraction and purification process, the real contents of coniferyl ferulate in stem and root should be higher than the detected values. In traditional Chinese medicinal practice, decoction of AS root is the main administration form. Whereas, previous work has shown that the decoction contains very low bioactive compounds (ligustilide, ferulic acid, coniferyl ferulate etc.), and hence might contributed little to the clinical efficacy, although many compounds are considered to possess multiple pharmacological activities [19]. Organic solvent can extract large amounts of bioactive compounds. However, it should be noted that a long-term preservation at room temperature of extracts might result in a significant degradation of coniferyl ferulate. In addition, ligustilides are easy to polymerize when exposed to sunlight. It was reported that ligustilide dimers and trimers had lower activity in inhibition of platelet aggregation. Hence, the extracts from Angelica or isolated ligustilides should be well stored to avoid the reduction of biological efficacy.

## 3. Materials and Methods

### 3.1. Materials and Chemicals

The whole plant of mature *Angelica sinensis* (AS) was collected from Min Country, Gansu province of China in mid-October. The stem (without leaves) and root were separated into two parts, and air-dried at 50 °C for three days. The samples were ground in a knife mill to collect the saw dust passing through a 2 × 2 mm^2^ sieve. The powdered samples were sealed in a plastic bag and stored at 2−5 °C for further analysis.

Cinnamic acid (99%), vanillin (99%), *p*-hydroxybenzoic acid (98%), protocatechuic acid (97%), ferulic acid (98%), caffeic acid (98%) and campesterol (98%) were purchased from Macklin Biochemical Co., Ltd. (Shanghai, China). *Z*-ligustilide (98%), Butylidenephthalide (98%), Senkyunolide I (98%), stigmasterol (98%), and *β*-sitosterol (98%) were purchased from Chroma Biotechnol. Co., Ltd. (Chengdu, China). Coniferyl ferulate (97%) was synthesized in a laboratory according to the literature [36], and the structure and purity were elucidated by 1H NMR (Figure S1). The HPLC-grade acetonitrile (≥99.9%) and methanol (≥99.9%) were bought from Merck KGaA (Darmstadt, Germany).

### 3.2. Isolation and Purification of Oily Extracts

Two gram AS stem and root samples were ultrasonic extracted with 50 mL methanol-formic acid (95:5, *v*/*v*) at 30 °C for 40 min, 2 times. The filtrates were collected and concentrated into ~2 mL under reduced pressure at 40 °C. In order to remove the soluble carbohydrates, the fluids were transferred to 50-mL centrifuge tubes charged with methanol (50 mL). After centrifugation, the supernatant liquids were concentrated and repeatedly precipitated with methanol, and the supernate was collected and concentrated into ~2 mL. Then, the samples were transferred into separatory funnel charged with 30 mL saturated ammonium chloride. The organics were extracted with ethyl acetate (3 × 30 mL). The combined organic fractions were then dried over anhydrous magnesium sulfate, filtered through a sand-core funnel, and evaporated by a rotary evaporator at 40 °C. Finally, 66 mg of oil product was obtained from the root sample and 36 mg of oil product was obtained from the stem sample.

### 3.3. GC–MS Procedure

#### 3.3.1. Derivatization and GC–MS Analysis

The isolated oily extracts were analyzed by GC–MS after trimethylsilyl (TMS) derivatization. The oil samples were dissolved into dichloromethane (concentration of 10 mg/mL), of which 1 mL solution was pipetted into a GC vial. Pyridine (50 μL) and *N*,*O*-bis trimethylsilyl trifluoroacetamide (BSTFA, 98%, 150 μL) were added into the vial, and the mixture was maintained at 60 °C for 40 min. The derivatized products were then analyzed by GCMS-TQ instrument (Shimadzu GCMS-TQ8040 triple quadrupole GC–MS/MS, Kyoto, Japan) equipped with an SH-Rxi-5Sil MS column (Shimadzu, 30 m × 0.25 mm × 0.25 μm, Kyoto, Japan). The program for GC–MS analysis is shown in Table S1.

#### 3.3.2. Qualitative Analysis

The reference compounds were dissolved in dichloromethane and diluted to five gradient concentrations to obtain the standard solutions. Then, a 1 mL solution was transferred into the GC vial and, after addition of pyridine (50 μL) and BSTFA (150 μL), the vial was maintained in an oven at temperature of 60 °C for 40 min. The standard solution was finally analyzed by GC–MS under the program of Table S1. The contents of analytes were determined by plotting the calibration curves with the peak areas versus the concentration of standard compounds.

### 3.4. HPLC–MS/MS Procedure

#### 3.4.1. Multiple Reaction Monitoring (MRM) for Coniferyl Ferulate Analysis

In order to eliminate the effects of impurities, the multiple reaction monitoring (MRM) model operated on a triple quadrupole LC–MS/MS (Shimadzu, LCMS–8050, Kyoto, Japan) was used to estimate the content of coniferyl ferulate. The method for MRM was established on Labsolutions LCMS Ver 5.6 according to LCMS–8050 Operators Guide (Shimadzu, Kyoto, Japan). The standard coniferyl ferulate solution (5 ppm) was analyzed by LC–MS/MS in both negative and positive ion modes. The most abundant ion with *m*/*z* of 193 (negative ferulate fragment) was chosen as precursor ion. The further fragment ions from the negative193 ion and corresponding fragmentor voltage were optimized on the build-in Optimization method. The optimized parameters for MRM model are shown in Table S2.

#### 3.4.2. Sample Analysis by LC–MS/MS

The purified oily extracts were dissolved in HPLC-grade methanol and diluted to 50 ppm. The sample solution was immediately filtered through a 0.22-μm polytetrafluoroethylene membrane and transferred into a LC vial for LC–MS/MS analysis in MRM model (Table S2).

The gradient concentrations (from 0.05 ppm to 10 ppm) of standard coniferyl ferulate and ferulic acid solutions were prepared in methanol (HPLC-grade). After being filtrated through a 0.22-μm polytetrafluoroethylene membrane, the standard solutions were immediately analyzed on LC–MS/MS in MRM model. The contents of ferulic acid and coniferyl ferulate in *Angelica sinensis* samples were calculated by plotting the calibration curves with the peak areas versus the concentration of the standard solutions. In order to investigate the degradation of coniferyl ferulate, the standard solution (1 ppm) was stored in the sample room (temperature 25 °C) of LC, and analyzed on MRM model after 1 h, 1.5 h, 9 h, 24 h, 48 h and 64 h.

## 4. Conclusions

Herein, the constituents of oily extract from *Angelica sinensis* (AS) stem were identified and compared with those in the root by GC–MS after trimethylsilyl derivatization or LC–MRM/MS. The stem contains lower contents of phthalides (*Z*/*E*-ligustilide, Senkyunolide I and *Z*-butylidenephthalide), ferulic acid, and coniferyl ferulate than the root; whereas, many other bioactive compounds were specifically identified in the extract of stem, including cinnamic, hydroxybenzoic, *p*-hydroxybenzeneacetic, vanillic and protocatechuic acids, 1-(4-hydroxyphenyl)-1, 2-ethanediol, and thymol-*β*-d-glucopyranoside. Moreover, there were more phytosterols (mainly *β*-sitosterol, with smaller amounts of campesterol and stigmasterol) in the stem than that in the root. The oily extract form AS stem potentially can be used as supplement for health food or therapeutic medicine.

## Figures and Tables

**Table 1:** Constituent identification from GC chromatograms of AS stem and root extracts.

Peak No.	RT (min)	Chemical Name	Formula	Relative Intensity of MS Ions	Stem	Root
1	17.46	Cinnamic acid	C_9_H_8_O_2_	220(27), 205(93), 161(100), 131(73), 103(62)	+	-
2	18.98	*p*-hydroxybenzoic acid	C_7_H_6_O_3_	282(23), 267(100), 223(98), 193(49), 126(13)	+	-
3	19.20	*p*-hydroxybenzeneacetic acid	C_8_H_8_O_3_	296(14), 252(23), 179(26), 164(18), 73(100)	+	-
4	19.60	Butylphthalide	C_12_H_14_O_2_	190(3), 172(3), 133(100), 105(33), 77(16)	-	+
5	20.00	*Z*-butylidenephthalide	C_12_H_12_O_2_	188(21), 159(100), 146(43), 131(42), 103(35)	+	+
6	20.92	*E*-butylidenephthalide	C_12_H_12_O_2_	188(21), 159(100), 146(43), 131(48), 103(39)	-	+
7	21.00	4-hydroxyphenyl-1, 2-ethanediol	C_8_H_10_O_3_	267(100), 193(7), 147(16)	+	-
8	21.16	*Z*-ligustilide	C_12_H_14_O_2_	190(55), 161(80), 148(100), 133(24), 105(70)	+	+
9	21.66	Vanillic acid	C_8_H_8_O_4_	312(44), 297(100), 282(36), 267(72), 253(48)	+	+
10	22.43	*E*-ligustilide	C_12_H_14_O_2_	190(54), 161(83), 148(100), 105(82), 77(36)	+	+
11	22.76	Protocatechuic acid	C_7_H_6_O_4_	370(37), 355(27), 311(25), 193(100), 165(7)	+	-
12	23.38	Myristic acid	C_14_H_28_O_2_	285(67), 145(19), 132(35), 117(100)	+	+
13	24.20	Senkyunolide G	C_12_H_14_O_3_	278(11), 249(93), 221(100), 205(22), 131(16)	+	+
14	25.32	Pentadecanoic acid	C_15_H_30_O_2_	299(74), 132(36), 129(37), 117(100)	+	+
15	25.48	Dibutyl phthalate	C_16_H_22_O_4_	223(5), 205(6), 149(100)	+	+
16	27.23	Palmitic acid	C_16_H_32_O_2_	328(5), 313(67), 132(42), 117(100)	+	+
17	27.26	Senkyunolide I	C_12_H_16_O_4_	368(19), 353(11), 252(100), 237(31), 223(19)	+	+
18	28.05	Ferulic acid	C_10_H_10_O_4_	338(100), 323(92), 308(74), 293(63), 249(52)	+	+
19	28.88	Caffeic acid	C_9_H_8_O_4_	396(54), 381(19), 307(8), 219(100), 191(14)	+	-
20	29.05	Heptadecanoic acid	C_17_H_34_O_2_	342(5), 327(71), 145(23) 129(41), 117(100)	+	+
21	29.27	Senkyunolide H	C_12_H_16_O_4_	368(20), 353(16), 252(100), 237(32), 147(45)	+	+
22	30.01	Mannonolactone	C_6_H_10_O_6_	319(14), 305(5), 229(7), 129(9), 73(100)	+	+
23	30.26	Linoelaidic acid	C_18_H_32_O_2_	352(4), 337(66), 262(44), 177(51), 73(100)	+	+
24	30.35	*Z*-oleic acid	C_18_H_34_O_2_	354(3), 339(47), 129(71) 117(98), 73(100)	+	+
25	30.52	*E*-oleic acid	C_18_H_34_O_2_	354(5), 339(83), 129(78), 117(100), 75(80)	+	+
26	30.82	Stearic acid	C_18_H_36_O_2_	356(7), 341(69), 132(42), 129(43)117(100)	+	+
27	32.55	Butyl 9,12-octadecadienoate	C_22_H_40_O_2_	336(6), 263(25), 178(18), 135(29), 109(42)	-	+
28	33.60	Eicosanoic acid	C_20_H_40_O_2_	384(10), 369(75), 132(44), 117 (100)	+	+
29	34.78	Thymol-*β*-d-glucopyranoside	C_16_H_24_O_6_	361(100), 271(23), 243(26), 169(40), 147(30)	+	-
30	35.18	Monopalmitin	C_19_H_38_O_4_	371(100), 239(27), 203(23), 147(36), 129(16)	+	+
31	35.75	Behenic acid	C_22_H_44_O_2_	412(12), 379(71), 132 (47), 117(100)	+	+
32	45.64	Campesterol	C_28_H_48_O	472(28), 382(83), 343(60), 255(28), 129(100)	+	-
33	46.30	Stigmasterol	C_29_H_48_O	484(25), 394(43), 255(41), 129(54), 83(100)	+	+
34	48.11	*β*-sitosterol	C_29_H_50_O	486(33), 396(88), 357(60), 255(29), 129(100)	+	+

“+” represents that the compound was detected; “-” represents that the compounds was not detected.

**Table 2:** Contents of 15 components in AS stem and root revealed by GC–MS.

Analytes	Stem		Root	
	mg/g Extracts	mg/g Stem	mg/g Extracts	mg/g Root
*Z*-butylidenephthalide	4.20 ± 0.31	0.074 ± 0.005	7.73 ± 0.78	0.26 ± 0.03
*E*-butylidenephthalide	nd ^1^	nd	4.86 ± 0.54	0.16 ± 0.02
*Z*-ligustilide	28.60 ± 1.24	0.51 ± 0.02	63.04 ± 3.06	2.08 ± 0.10
*E*-ligustilide	4.16 ± 0.10	0.076 ±0.002	10.94 ± 0.47	0.36 ± 0.02
Senkyunolide I	11.53 ± 3.21	0.21 ± 0.06	34.30 ± 8.50	1.13 ± 0.28
Senkyunolide H	nd	nd	17.71 ±0.37	0.58 ± 0.02
Cinnamic acid	3.15 ± 0.88	0.057 ± 0.018	nd	nd
*p*-hydroxybenzoic acid	3.48 ± 0.13	0.064 ± 0.002	nd	nd
Vanillic acid	1.16 ± 0.12	0.021± 0.002	nd	nd
Protocatechuic acid	3.00 ±0.26	0.055 ±0.004	nd	nd
Caffeic acid	2.00 ±0.15	0.036 ±0.002	nd	nd
Ferulic acid	7.10 ± 0.56	0.13 ± 0.01	18.18 ± 0.53	0.60 ± 0.02
Campesterol	4.93 ± 0.12	0.088 ±0.002	nd	nd
Stigmasterol	14.03 ± 0.41	0.26 ± 0.01	3.94 ± 0.18	0.13 ± 0.01
*β*-sitosterol	56.21 ± 1.20	1.01 ± 0.02	11.21 ± 0.75	0.37 ± 0.03

Values (mean ± SD) obtained from duplicate technical runs; ^1^ nd = not detected.

**Table 3:** Contents of ferulic acid (FA) and coniferyl ferulate (CA-FA) analyzed by LC–MS/MS on MRM mode.

Analytes	Stem		Root	
	mg/g Extract	mg/g Stem	mg/g Extract	mg/g Root
Ferulic acid	5.42 ± 0.65	0.098 ± 0.010	17.57 ± 0.91	0.58 ± 0.03
Coniferyl ferulate	1.16 ± 0.26	0.021 ± 0.004	15.76 ± 0.03	0.52 ± 0.01

Values (mean ± SD) obtained from duplicate technical runs.

## Supplementary Materials

The following are available online, Figure S1: ^1^H NMR of coniferyl ferulate, Figure S2: Linear regression equations for calculation of bioactive compounds identified in GC chromatograms, Figure S3: MS spectrum of coniferyl ferulate (CA-FA) (a) and the optimized MS fragmentations and corresponding optimal collision energys (CE) for [FA − H]^−^ (*m*/*z* 193) ion in MRM mode (b), Figure S4: Linear regression equations of ferulic acid (FA) and coniferyl ferulate (CA-FA) in LC chromatograms, Figure S5: Degradation of coniferyl ferulate (CA-FA) in methanol solution with time, Table S1: GC–MS program for analyzing derivatized extracts of *Angelica sinensis*, Table S2: LC–MS/MS program for analysis of coniferyl ferulate and ferulic acid.
