# Supplementary material for: Angelica Stem: A Potential Low-Cost Source of Bioactive Phthalides and Phytosterols
Source: Molecules. 2018 Nov 23;23(12):3065. doi: 10.3390/molecules23123065 (PMC6321507; doi:10.3390/molecules23123065)
Supplement: Supplementary file 1 [file molecules-23-03065-s001.pdf]

Angelica Stem: A Potential Low-cost Source of Bioactive Phthalides and Phytosterols

Chengke Zhao [1], Yuan Jia [1] and Fachuang Lu [1, 2,*]

[1] State Key Laboratory of Pulp and Paper Engineering, South China University of Technology, Guangzhou, 510640, China; mszck@foxmail.com (C. Zhao); 201620121278@mail.scut.edu.cn (Y. Jia); fefclv@scut.edu.cn (F. Lu)

[2] Guangdong Engineering Research Center for Green Fine Chemicals, Guangzhou 510640, China

* Correspondence: fefclv@scut.edu.cn (F. Lu); Tel.: +86-020-8711-3953

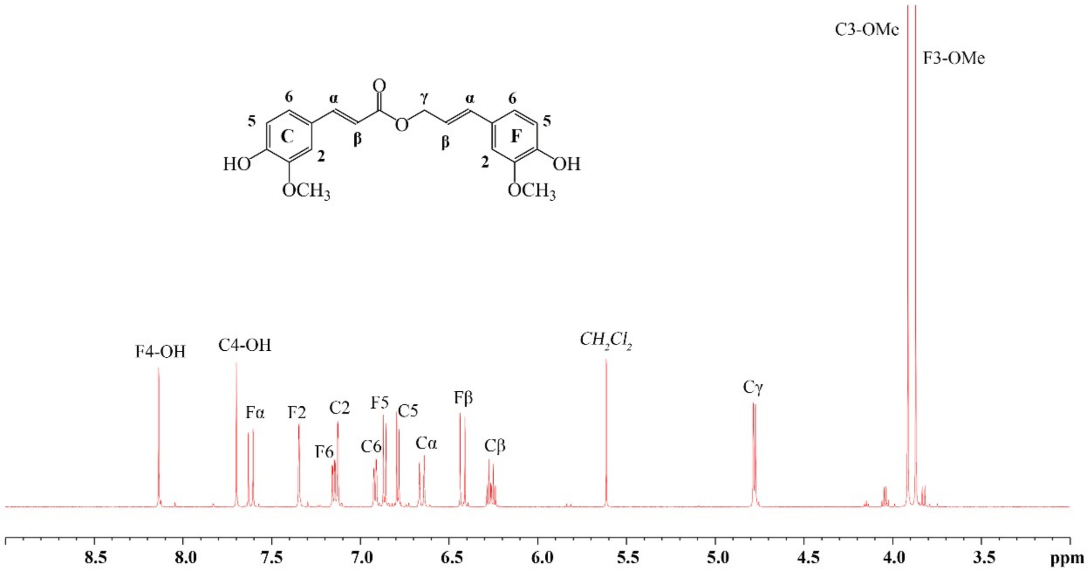

Figure S1. ^{1}H NMR of coniferyl ferulate.

Table S1. GC-MS program for analyzing derivatized extracts of angelica sinensis.

Gas Chromatograph	GC-2010 Plus
Inlet	200 °C<br>Split liner with glass wool (Shimadzu 220-90784-00)<br>Split injection (20:1)
Column	SH-Rxi-5Sil MS 30 m x 0.25 mm x 0.25 µm (Restek 13623)<br>Helium carrier gas<br>Constant linear velocity 47.2 cm/sec
Oven Program	50 °C, ramp 8 °C/minute to 150 °C, 5 °C/minute to 230 °C, 10°C/minute to 280 °C, hold 20 minutes<br>MS interface 280 °C<br>Analysis time 55 minutes
Mass Spectrometer	GCMS-TQ8040
Ion Source	200 °C<br>Electron ionization (EI) mode, 70 eV
Operation Mode	Q3 scan
Detector	Electron multiplier 1.6 kV

Table S2. LC-MS/MS program for analysis of coniferyl ferulate and ferulic acid.

LC Chromatograph	Nexera UHPLC system
Injector	SIL-20AXR<br>Inject 1μL
Pump	Binary high-pressure gradient system<br>Mobile phase: Acetonitrile-aqueous (with 0.1% acetic acid)<br>Total flow rate 0.4 mL/min
Time program	0-2 min 5% acetonitrile; 2-6 min linear gradient to 40% acetonitrile; 6-10 min linear gradient to 80% acetonitrile; 10-15 min isocratic elution 80% acetonitrile; 15.1 -18 min 5% acetonitrile.
Column	Shim-pack GISS C18 Column SH-Rxi-5Sil MS 50 mm x 2.1 mm x 1.9 μm
Oven Program	CTO-20AC, 30 °C
Mass Spectrometer	LCMS-TQ8050
Interface	Electronic Spray Ion (ESI) mode<br>Gasl 3 L/min; drying gas 10 L/min<br>Interface 200 °C; DL250 °C; Heating block 300 °C
Operation Mode	Multiple Reaction Monitoring (MRM), negative ion mode<br>Argon gas, 17 kPa<br>Ion range 80-500 (m/z)<br>Event time 0.309 s; total measure time 15 min

MRM Transition Details

Compound Name	Retention time (min)	Transition 1	CE 1	Transition 2	CE 2	Transition 3	CE 3
Ferulic acid	5.12	193>178.2	14	193>134.1	14	193>133.1	25
Coniferyl ferulate	8.04	193>178.2	14	193>134.1	14	193>133.1	25

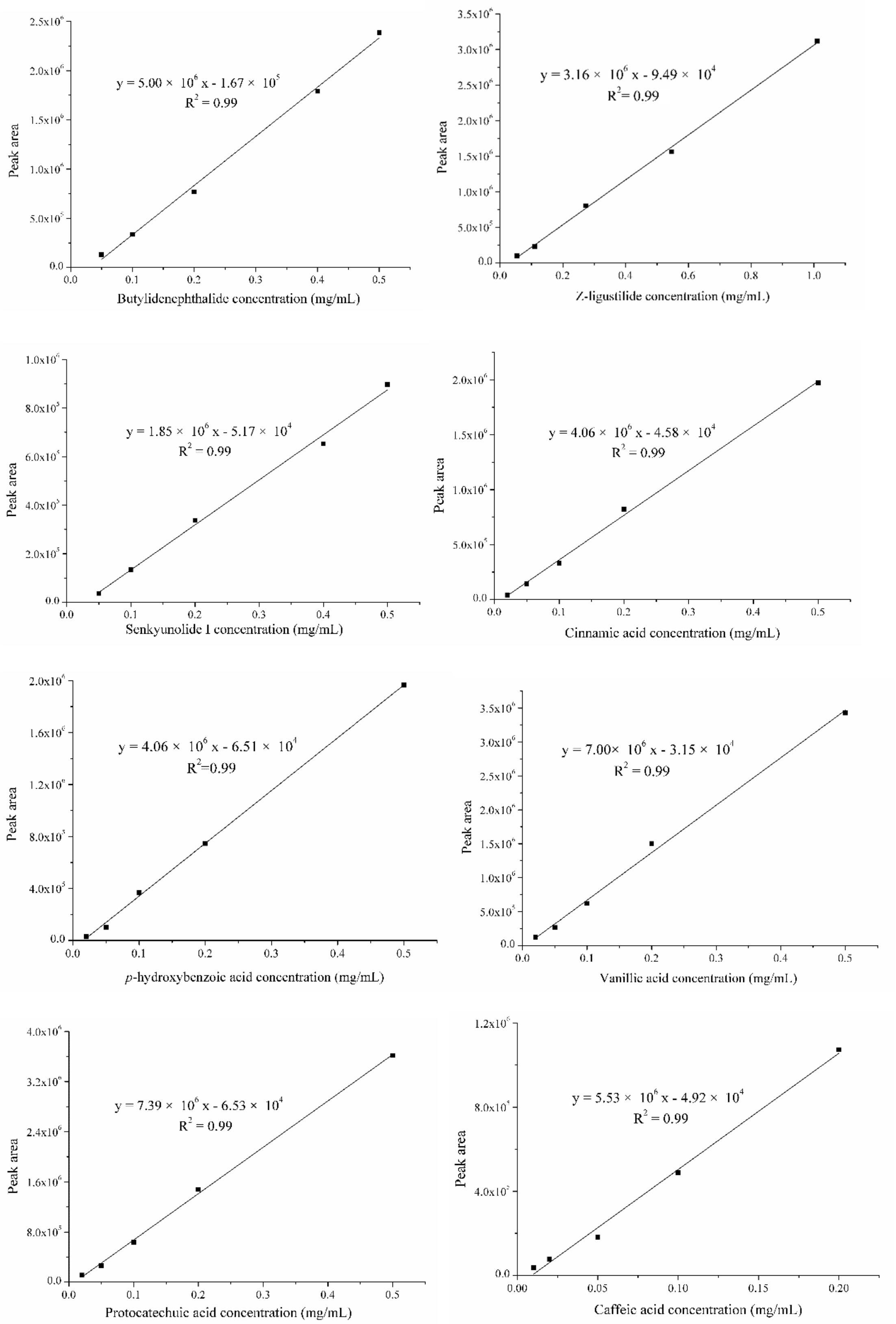

Figure S2. Linear regression equations for calculation of bioactive compounds identified in GC chromatograms (continued).

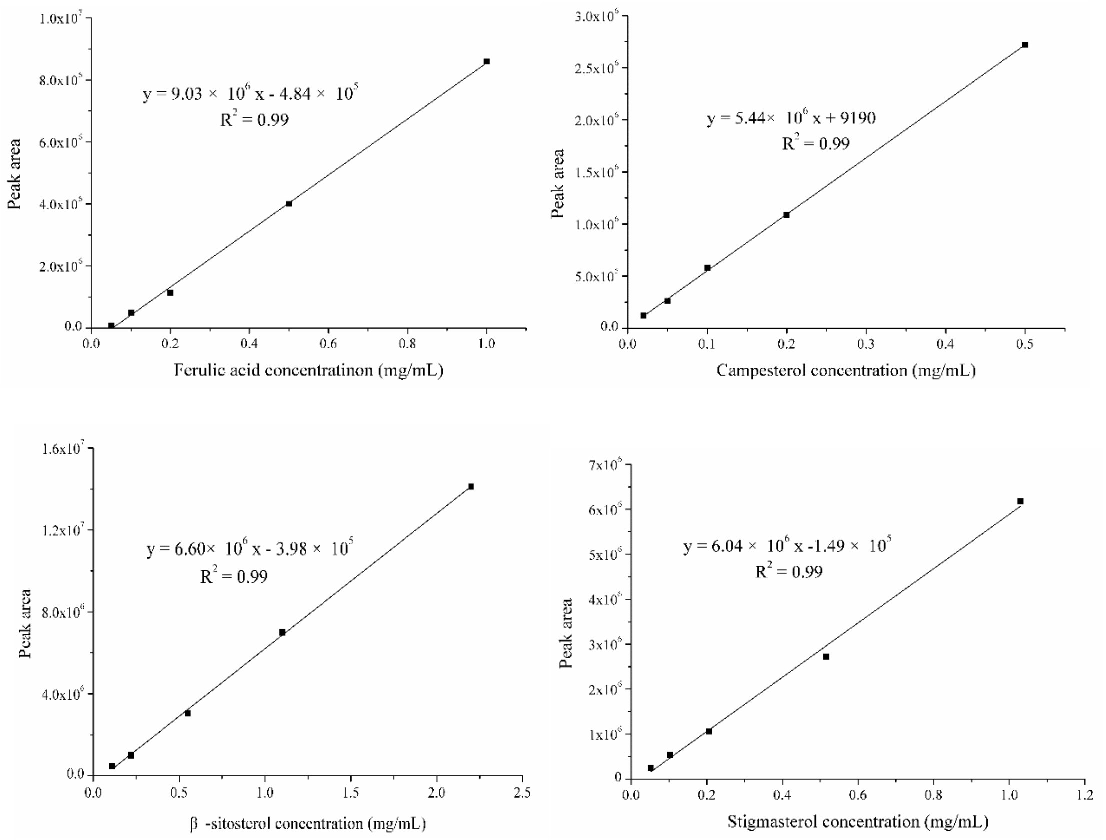

Figure S2. Linear regression equations for calculation of bioactive compounds identified in GC chromatograms.

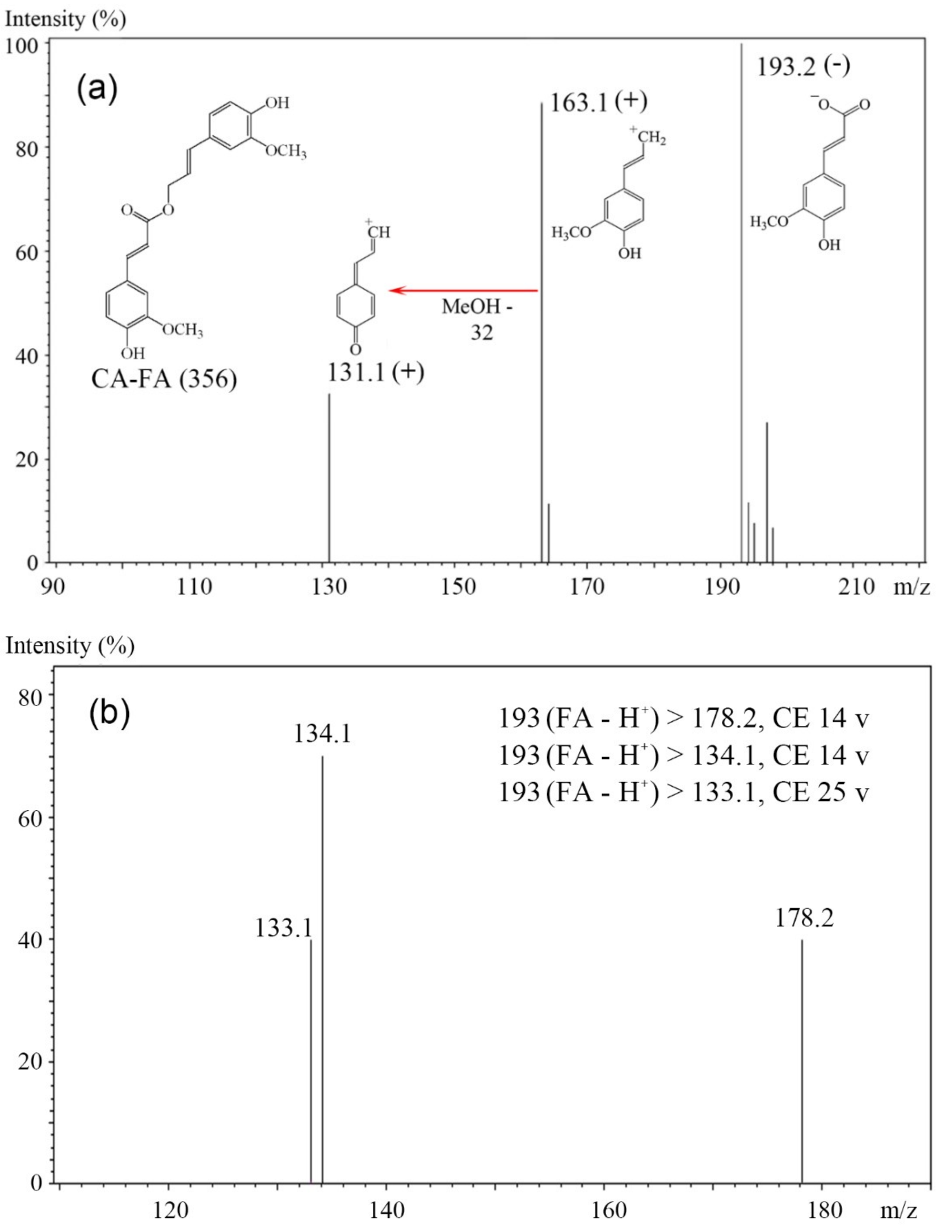

Figure S3. MS spectrum of coniferyl ferulate (CA-FA) (a) and the optimized MS fragmentations and corresponding optimal collision energys (CE) for $[FA-H]^-$ (m/z 193) ion in MRM mode (b).

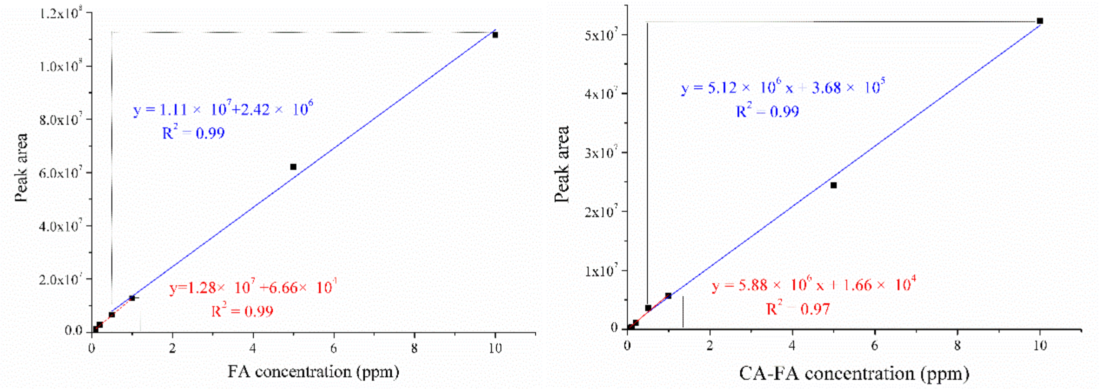

Figure S4. Linear regression equations of ferulic acid (FA) and coniferyl ferulate (CA-FA) in LC chromatograms.

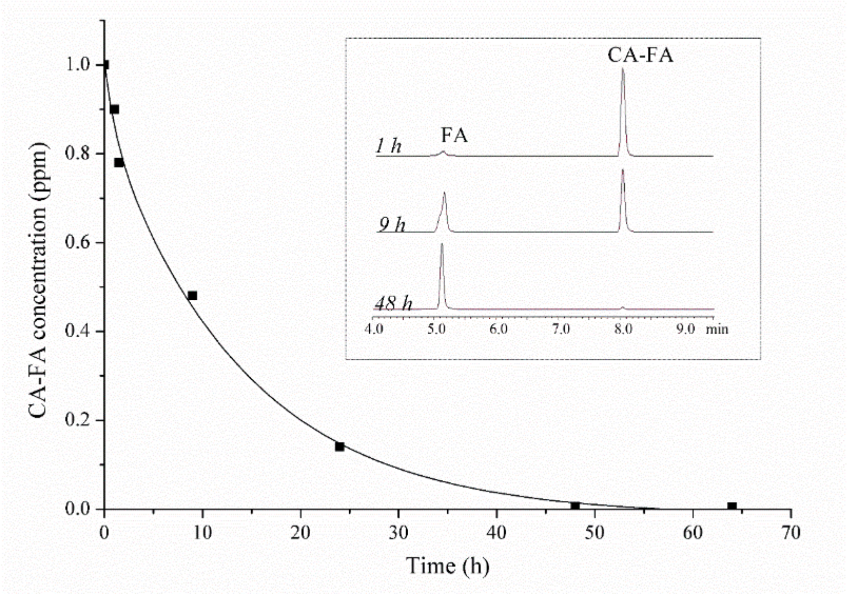

Figure 5. Degradation of coniferyl ferulate in methanol solution (temperature 25 °C). FA is ferulic acid, CA-FA is coniferyl ferulate.
